# Racing Demands of Off-Road Triathlon: A Case Study of a National Champion Masters Triathlete

**DOI:** 10.3390/sports9100136

**Published:** 2021-09-30

**Authors:** Christopher R. Harnish, Hamish A. Ferguson, Gregory P. Swinand

**Affiliations:** 1 Department of Exercise Science, Murphy Deming College of Health Sciences, Mary Baldwin University, Fishersville, VA 22939, USA; 2 Centre for Bioengineering, Department of Mechanical Engineering, University of Canterbury, Christchurch 8041, New Zealand; hamish.ferguson@pg.canterbury.ac.nz; 3 London Economics, London WC2R 1LA, UK; gswinand@londoneconomics.co.uk

**Keywords:** XTerra, trail running, critical power, MTB, off-road cycling economy, cross triathlon

## Abstract

(1) Background: This report examines the unique demands of off-road triathlon (XT) by presenting physiological, field, and race data from a national champion off-road triathlete using several years of laboratory and field data to detail training and race intensity. (2) Methods: Laboratory and field data were collected when the athlete was at near peak fitness and included oxygen consumption (VO_2_), heart rate (HR), power output (W), and blood lactate (BLC) during cycling and running, while HR, cycling W, and running metrics were obtained from training and race data files over a period of seven years. Intensity was described using % HR max zones (Z) 1 < 75%, 2 = 75–87%, and Zone 3 > 87%, and W. An ordinary least squares analysis was used to model differences between event types. (3) Results: Weather conditions were not different across events. XT events had twice the elevation change (*p* < 0.01) and two-three times greater anaerobic work capacity (W’) (*p* < 0.001) than road triathlon (ROAD), but similar HR intensity profiles (max, avg, and zones); both events are predominately performed at >Z2 or higher intensity. Championship XT events were longer (*p* < 0.01), with higher kJ expenditure (*p* < 0.001). Ordinary Least Squares (OLS) modelling suggested three variables were strongly related (R^2^ = 0.84; *p* < 0.0001) to cycling performance: event type (XT vs ROAD), total meters climbed, and total bike duration. Championship XT runs were slower than either regional (*p* < 0.05) or ROAD (*p* < 0.01) runs, but HR intensity profiles similar. OLS modelling indicates that slower running is linked to either greater total bike kJ expenditure (R^2^ = 0.57; *p* < 0.001), or total meters gained (R^2^ = 0.52; *p* < 0.001). Race simulation data support these findings but failed to produce meaningful differences in running. Conclusions: XT race demands are unique and mirror mountain bike (MTB) and trail running demands. XT athletes must be mindful of developing anaerobic fitness, technical ability, and aerobic fitness, all of which contribute to off-road cycling economy. It is unclear whether XT cycling affects subsequent running performance different from ROAD cycling.

## 1. Introduction

The sport of triathlon is characterized by successive swimming, cycling, and running, and events can be as short as 1-h or as-long-as 8-h for elite competitors. While each distance category may present significantly different physiological demands on athletes [1], off-road triathlon (XT), aka XTerra triathlon [2], presents the unique demands of mountain biking (MTB) and running on technical trails with substantial ascents and descents. Additionally, while Championship races are ~1500-m swim, 30-km bike, and 10-km run, and regional races range from 800 to 1200-m swims, 16 to 30-km bike, and 5 to 10-km run, considerable variation exists across races and venues. For example, some races may include both urban and forested courses, while others take place in the deep forest with very technical trails. Thus, preparation for XT may be very challenging.

The physiological and skill requirements of MTB can differ considerably from ROAD cycling, with factors such as anaerobic power and capacity, technical ability and overall off-road cycling economy being as important as aerobic fitness [3,4]. Likewise, trail running requires greater skill and poses greater physiological and mechanical stress [5,6,7,8]. While Lepers and Stapley have reported age-related [7] and gender-related [9] differences in road triathlon (ROAD) versus XT performances, little empirical data is available for athlete characteristics or race demands. The exercise-intensity profile and race characteristics of XT can aid in the design of training programs and race strategy. Furthermore, preparatory races are often used in the build-up to goal races, making the exercise-intensity profile helpful for understanding the training load imposed on athletes. 

In this case report we present extensive physiological, field testing, and race data, from a master’s national champion XT athlete, to demonstrate the unique demands of XT compared to ROAD. We hypothesize XT will elicit unique physiological and mechanical (e.g., power output, kJ expenditure) exercise-intensity profiles. We also expect that the unique race demands of XT should adversely impact running performance. We believe, when adjusted for duration, the intensity-profile and event characteristics of Championship-level events will be significantly higher than non-championship events. Finally, it is hypothesized, total bike kJ expenditure, bike anaerobic energy contribution, course elevation gain, and total run duration would be significant determinants of running performance. 

## 2. Materials and Methods

### 2.1. Participant Information 

This case study adhered to Mary Baldwin University IRB policies. Written consent was obtained from a 46 year accomplished male triathlete (175 cm, 64 kg, VO_2 Max_ 4.15 L·min^−1^) who has competed for 11-year, with two age-group national championship podiums, three top-5 finishes in either long course duathlon and XT triathlon, and an age group national championship off-road triathlon. Prior to triathlon, the athlete competed as an U.S. Category 1 road cyclist for 10-year (60.9 kg, VO_2 Max_ 4.52 L^·^min^−1^). Detailed training information on this athlete can be found in Supplemental Table S1, while Supplemental Figure S1 presents HR TRIMP training load calculated based on a simplified 3-phase TRIMP score [10].

### 2.2. Equipment 

Laboratory data collection included expired respiratory gases measured using a Parvo TrueMax metabolic cart (Parvo Medics, Salt Lake City, UT, USA), while lab and field blood lactate samples (0.7 µL) were collected from the finger and analyzed using Lactate Plus analyzer (Nova Biomedical, Waltham, MA). All road bicycle power data were measured using a Power2Max (P2Mr) NGeco power meter (Power2Max, North Vancouver, CA, USA), while all XT bicycle data were measured using a Power2Max S-type power meter (P2Mm). Heart rate (HR) was measured with a Wahoo TickrX HR belt, while running metrics, including power output, were measured using a Stryd 3rd generation footpod (Stryd, Boulder, CO, USA). All data were collected and stored using a Garmin Fenix 5 watch (Garmin International, Olathe, KS, USA).

### 2.3. Physiological Testing 

The athlete had maintained detailed testing records dating back to 1993, including standardized lab testing from 2014, until the present. We employed the same testing protocols in our report to maintain consistency. The athlete was tested in the lab during the winter, and again in early spring, 2-weeks prior to field race simulations, completing sub-max run testing, resting 30-min before completing bicycle testing, then immediately repeating run testing. 

### 2.4. Bicycle Testing 

VO_2 Max_ testing was conducted on the athlete’s own bicycle attached to a Wahoo Kickr Core trainer. The athlete used a freely chosen cadence above 85 rpm, and testing started at 140 W, increasing 40 W every 5 min. The test was terminated upon voluntary exhaustion. Blood lactate samples were collected at the end of each stage. 

### 2.5. Run Testing 

Run testing was performed on a NordicTrack X11i motorized treadmill (Logan, UT) initially set at 1% incline. All treadmill testing began with a 5-min easy warm-up at 10.5 kph, increasing 1.6 kph every 5-min until reaching 16.1 kph, after which gradient was increased every minute until voluntary exhaustion. Blood lactate samples were collected at the end of each stage while the athlete briefly stopped (~30-s). 

### 2.6. Simulated Race Tests

Two field tests were completed 4-days apart to better compare the effects of Road, then XT cycling on run performance. All testing was staged in the same location. Owing to dangerous water conditions, a matched submaximal 5-km run was used prior to each ride (23:51 vs. 23:46). The RT course (43.1-km, ↑ 233-m) was rolling and used parts of the 2015 UCI World Championship team time trial course, while the XT course (24.8-km, ↑ 329-m) used the XTerra East Championship course. The 10-km run consisted of an out-and-back mixed surface course. Weather conditions were similar for both trials, and the athlete adhered to a nearly an identical training and meal plan 24-h prior to testing, and ingested similar fluid (1200 mL) and carbohydrate (155 g) amounts. Blood lactate and RPE were taken after each leg.

### 2.7. Race Data

Twenty-three races spanning 7-years of competition were used in the analysis and included two national and four regional XT championships—1.6-km swim, 30-km bike, 10-km run (CXT); six consecutive years of the same regional XT—~1.0-km swim, 23-km bike, 8-km run, plus five other races (RXT); and five road events (ROAD) that included two duathlon national championships at the same venue (10-km run, 50-km bike, 10-km run). These races were chosen because HR, power output, and run metrics were all recorded during the bike and run segments. Owing to wide variations in race formats for swim distance and timing, minimal swim data are presented.

### 2.8. Data and Statistical Analyses

Data are expressed as mean ± SD. Normalized power (NP^®^) [10] was used to mitigate variations in power due to terrain. Critical power (CP), and anaerobic work capacity (W’) [11] were obtained from training data using Golden Cheetah v 3.5 software (www.goldencheetah.org). VO_2 Max_ was defined as the highest 1-min average achieved during testing. Running economy at 4 mM·L^−1^ was calculated as mlO_2_·kg^−1^·m^−^HR zones were determined based on blood lactate at 2 mM·L^−1^ (Z1) and 4 mM·L^−1^ (Z2), and above 4 mM·L^−1^ (Z3) [12]. From the two tests, training zones were estimated as zone 1 < 75%, Zone 2 = 75–87%, and zone 3 > 87% maximum HR, respectively [13].

To better contextualize the differences between event types an ordinary least squares analysis was run using Stata [14] (StataCorp. Stata Statistical Software: Release College Station, TX: StataCorp LLC) with a significance level of 0.05, and adjusted R^2^ (R^2^) reported for modelling. The strongest relationships from the modelling were then presented. Complete output reports are presented in Supplemental Table S2.

## 3. Results

### 3.1. Physiological and Training Data

Cycling CP was 290 W and W’ 23.9 kJ. Running at 4 mM·L^−1^ the athlete’s pace was 4:09 (min·km^−1^), HR was 159 bpm, and economy was 209 mlO_2_·kg^−1^·m^−^Training averaged 10.3 + 1.0 h·week^−1^ and was distributed as 83.0 + 2.0% Z1, 13.6 + 2.0% Z2, and 3.4 + 1.0% ZThere were no significant differences (*p* > 0.10) in age, critical power, or 4 mM run pace between CXT, RXT, or ROAD events; i.e., the athlete was at a similar fitness level across races. As noted above, quality swim data were difficult to obtain so only time and stroke rate are presented in Table 1.

### 3.2. Race Data Analysis

Race and field data are summarized in Table 1 There were no significant differences in temperature (*p* > 0.13) or humidity (*p* > 0.21). CXT bike durations were significantly higher than RXT (*p* = 0.0003) or ROAD (*p* = 0.001), as well as significant differences in NP RXT (*p* = 0.0456), NP ROAD (0.0136), kJ expenditure RXT (*p* = 0.0005), and kJ expenditure ROAD (*p* = 0.0124). However, other differences highlight some of the unique challenges of XT events. For example, when adjusted for time, CXT (*p* = 0.0033) and RXT (*p* = 0.0039) had more than double the amount of elevation change than ROAD (2.0 ± 1.6 m·min^−1^). Even after adjusting for the elevation gain, CXT NP was significantly lower than ROAD (*p* = 0.005), but not RXT (*p* = 0.186). The elevation changes along with the stochastic nature of MTB may account for the 2 to 3-fold higher W’ expenditure for both CXT (*p* < 0.0001) and RXT (*p* = 0.0005) as compared with the ROAD (59.6 ± 22.0 kJ). However, adjusted elevation and W’ were co-variants, suggesting course elevation characteristics may be more important. In contrast, the maximal and average HR were similar across all bike legs.

Running data showed less differentiation between events, with the most notable being CXT runs were significantly longer than either RXT (*p* = 0.0027) or ROAD (*p* = 0.0017) runs. Running legs were significantly slower for CXT compared to ROAD (p=0.0029) and RXT (0.0444), as well as RXT and ROAD (*p* = 0.0037). This slower off-road running speed was likely the result of a significantly shorter stride length for CXT (*p* = 0.0004) and RXT (*p* < 0.0001). Average HR were similar across all run events, while maximal HR was significantly lower (*p* = 0.0110) during RXT when compared to ROAD. 

The overall (i.e., bike and run) intensity profile using HR zones were similar with the exception that time in zone 2 was significant higher in CXT compared to RXT (*p* = 0.0034) and ROAD (*p* = 0.0102), but not between RXT and ROAD (*p* = 0.4126).

To further study the impacts of event and pacing profiles on energy output intensity, we conducted regression analysis using both bike and run intensity measures as the dependent variables. The model estimated for cycling was:

(1) $$\mathrm{kJ}\_\min_{bike}=\alpha_{i}\pm \beta elev_{bike}\pm \gamma time_{bike}\pm \varepsilon$$

where kJ_min is the work output rate measured from a power meter, *α_i_* is a set of dummy variables for the event type, RXT, CXT, Road, elev is the positive elevation change and time is bike leg duration; the Greek letters are parameters to be estimated, including *ε* a classical random error term.

OLS modelling for cycling suggested three variables were strongly related to performance, (kJ·min^−1^) (R^2^ = 0.84; *p* < 0.0001): event type (CXT, RXT, ROAD), total meters climbed, and total bike duration. The coefficient estimates of the model suggest ROAD events have a statistically significant higher work output rate than XT events, but the greater the elevation gain, the greater the work rate in kJ·min^−^In contrast, the longer the event, the lower the kJ·min^−1^.

Based on the sample of races examined, OLS regression model results were less clear for running. Running pace was used as the performance measure. Adjusted elevation gains (m·min^−1^ run time) were not different across events, and likely covaried with total run time, thus total meters gained were used for the analyses. When looking at run performance by itself, event type (XT vs. ROAD) and total meters gained were most strongly related (R^2^ = 0.52; *p* = 0.0009) to slower run pace (min·km^−1^). Similarly, depending on the event type and greater total kJ expenditure on the bike, the slower the running pace (R^2^ = 0.57; *p* = 0.0004). However, combining the three aforementioned variables resulted in a slightly worse fitting model (R^2^ = 0.52; *p* = 0.0019).

### 3.3. Field Testing Data Analysis

**Figure 1:**
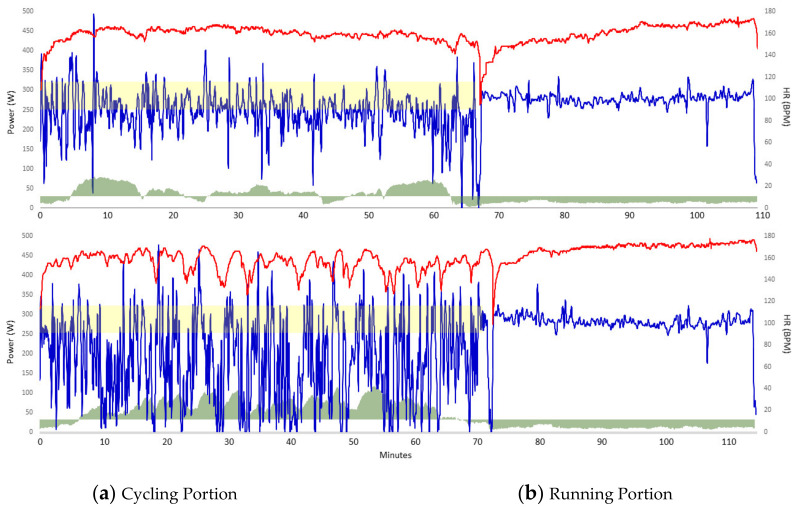
Comparison between RT (Top) and XT (Bottom) field testing of similar cycling duration (66.9 vs 70 min, respectively); Power is depicted in blue (x-axis), HR red (y-axis), and elevation changes are illustrated in green. Note the large variations in power and HR during XT and relative W’ (15.9% vs 6.1% total kJ). The yellow highlight represents the ± 10% of critical power (290 W).

As noted earlier, field data are summarized in Table 1 Overall, data indicate course characteristics and power output differed significantly between bike legs, run data were remarkably similar. Figure 1 are graphic comparisons between matched XT and RT field tests. These data illustrate the high stochasticity of the MTB leg of XT, resulting in greater HR variations and neuromuscular load. Nonetheless, running data were similar, with XT run time only 27 s (1.1%) faster.

## 4. Discussion

The purpose of this case report was to examine the physiological and mechanical characteristics of off-road versus, road triathlon events using several years of data. We hypothesized that XT would elicit unique physiological and mechanical exercise-intensity profiles, and the unique race demands of XT will adversely impact running performance. In addition, we believed the intensity-profile and event characteristics of Championship-level events would be significantly higher, independent of duration. Our findings indicate, however, despite significantly greater elevation changes and greater anaerobic work demands (W’), the HR intensity profiles (e.g., max, avg, and zones) were remarkably similar across all events. While CXT events were longer, with higher kJ expenditure, the work rate was lower. We also hypothesized that total bike kJ expenditure, bike W’, course elevation gain, and total run duration would be significant determinants of running performance. While XT runs were significantly slower than ROAD, the HR intensity profiles were similar across all events. The major influences on running outcome were total kJ expenditure on the bike and the total elevation gain during the run.

Our race data from CXT and RXT demonstrate both events present unique challenges on the athlete despite presenting similar HR zone intensity profiles. Of note, adjusted elevation gains (m·min^−1^) and mechanical demands appear greater. For example, CXT and RXT W’ were 15.7% and 18.0% of total kJ expenditure, respectively, compared to 7.1% ROAD. MTB performance relies on technical skill, as well as both anaerobic and aerobic fitness which contribute to an overall off-road cycling economy. Differences between bike legs were further highlighted by field data, clearly showing differences in power stochasticity and even HR fluctuations, despite similar HR zone profiles [3,4,12].

Unsurprisingly, the longer the event, the greater the kJ expenditure, but also the lower the NP; i.e., longer events are paced at a lower power. ROAD events, which were of similar duration to RXT, showed a higher work rate than either RXT or CXT. However, CXT events showed a lower work rate than RXT, even after adjusting for time and elevation gains, suggesting that other factors are affecting pacing during longer CXT events. The underlying mechanisms for pacing during endurance events are beyond the scope of this case report, but suggest in long duration events (> 90-min), that momentary rating of perceived exertion (RPE) and anticipatory exertion can influence pacing early on [15]. While body temperature and fuel availability influence pacing in the latter part of the event [15,16]. Athletes will also anticipate a required pace for an event, and often adopt a positive (i.e., slowly declining pace over time), with pace decreasing more significantly over longer durations [16,17], particularly if early maximal efforts (i.e., above CP) are employed; the latter of which was routinely employed by this athlete in XT events. It has also been suggested that the longer the duration, the more difficult overall pacing becomes and may result is overly conservative pacing to conserve energy (S. Marcora, personal communication, July 15, 2021). These data suggest the greater decline in work rate in CXT events may be the result of a positive pacing strategy and “clamped” RPE at a specific exertion level [15].

As expected, resulting running data across events were slower for off-road events from a shorter stride length, and specific terrain characteristics, most notably elevation gain [18]. HR intensity profiles were also similar. It is noteworthy that the greater the kJ expenditure on the bike, the slower run times were, regardless of terrain. Prior research has shown variations in cycling power and/or cadence may decrease [19], or improve [20] running performance, specific cadence/power strategies, high or low, could result in better run performance [21], and bike-run performance is variable among triathletes, but is also a significant predictor of triathlon success [1,22,23,24,25,26]. Interestingly, XT power stochasticity and W’ expenditure did not relate to slower run times. Moreover, field test data did not bear this out, with the XT test run being slightly faster. It must be noted, the run course used in our field tests included both road and trail sections but was not technical. It is unclear if a more challenging run terrain [6,7,8,18] would be impacted by XT differently.

### Applications and Limitations

This case report provides unique insight into off-road triathlon that can help guide future research, training development, and race strategy. Unsurprisingly, the bike demands for XT are similar to MTB race demands [3,27], showing wide power variations and nearly 3-fold greater W’ which may not be reflected in HR profiles. However, like ROAD, total kJ expenditure was a major determinant of run performance. Therefore, coaches and athletes should first and foremost optimize off-road cycling economy similar to MTB cyclists. This is particularly important for both individual bike performance and to minimize kJ expenditure, which was shown to negatively impact running performance. The W’ data also indicate that XT athletes should include anaerobic and neuromuscular training for cycling [3,27]. While overall run training likely does not differ from ROAD, research on trail running indicates that some specific preparation is also necessary to optimize XT run performance. Finally, our field testing indicates that such sessions could be useful in preparing for or predicting upcoming races without the need of competition.

This report, and its conclusions, are born from a single athlete and the results may not generalize across populations and the statistics used here are intended to help contextualize the data. It should also be noted that we have not discussed the impact terrain has on fueling and hydration needs during events. However, we believe the range of event data will help guide coaches, athletes, and perhaps researchers in the further study and optimization of off-road triathlon.

## 5. Conclusions

XT race demands are unique and mirror MTB and trail running demands, thus differing significantly from ROAD. XT bike sections result in much larger fluctuations in power and HR relative to ROAD, with a large anaerobic energy contribution and likely neuromuscular load and a lower overall work rate when controlling for length and elevation change. Thus, XT athletes must be mindful of developing anaerobic fitness, technical ability, and aerobic fitness, which all contribute to off-road cycling economy. Additionally, XT courses vary widely in technical and terrain aspects, making even pacing impossible. Athletes need a specific training and racing approach to address the unique physiological and technical aspects in training, as well as examine race course features to develop race and feeding strategies.

## Figures and Tables

**Table 1:** Summary data for race XT Championships, XT Regional, Road events, as well as two matched road and XT field tests. Swim data are presented for context.

				Field Testing	
Event Type	CXT	RXT	ROAD	Road	XT
Temperature (^o^F)	77.7 ± 12.1	71.5 ± 7.1	70.4 ± 4.4	60	73
Humidity (%)	72.5 ± 19.3	71.6 ± 17.9	80.0 ± 18.0	89	83
Total Duration (min)	194.7 ± 21.5	127.1 ± 9.2^#^	118.9 ± 35.4^#^	132.1	135.2
**Total Zone Minutes**					
Z1	11.6 ± 7.6	21.2 ± 18.5	16.7 ± 22.1	4.0	15.5
Z2	117.1 ± 28.8	67.3 ± 12.3	63.9 ± 32.2	75.0	61.4
Z3	38.6 ± 25.6	20.3 ± 20.4^#^	35.9 ± 32.9^#^	29.9	47.5
**Swim Data**					
Time (min)	24.7 ± 7.8	17.7 ± 6.2	17.7 ± 4.7	N/A	N/A
Stroke Rate (SPM)	32.0 ± 1.9	31.9 ± 2.2	31.3 ± 2.3		
**Bike Data**					
Time (min)	111.3 ± 16.0	64.5 ± 6.3^#^	61.8 ±19.3^#^	70.0	66.9
Average KPH	17.0 ± 2.9	20.6 ± 0.7^#^	37.7 ± 0.1^#$^	38.7	20.2
NP (W)	216.3 ± 6.7	231.2 ± 2.4	227.0 ± 8.5^#^	252.0	223.0
Cadence (RPM)	78.3 ± 1.8	74.6 ± 8.1	94.0 ±4.2^#$^	92.0	85.0
kJ expediture	1183.3 ± 167.5	736.8 ± 58.5^#^	842.8 ± 223.3^#^	981.0	804.0
W’ (kJ)	184.0 ± 31.7	132.4 ± 9.9^#^	59.6 ± 22.0^#$^	67.0	126.0
Peak HR (bpm)	169.3 ± 4.5	169.4 ± 3.0	172.0 ± 3.8	168.0	171.0
Average HR (bpm)	154.5 ± 3.9	157.0 ± 3.1	161.0 ± 4.5	159.0	156.0
Elevation Gain (m)	594.1 ± 104.0	328.8 ± 102.6^#^	145.8 ± 120.3^#$^	221	311
Adjusted Gain (m·min^−1^)*	5.4 ±0.7	5.1 ± 1.5	2.0 ± 1.6^#$^	0.5	0.5
Blood Lactate (mM)	--	--	--	4.2	5.9
RPE	--	--	--	8	8
**Run Data**					
Run Pace (min·km^−1^)	5.25 ± 0.60	4.72 ± 0.35	4.22 ± 0.31^#$^	4.29	4.24
Steps·min^−1^	169.7 ± 5.7	170.8 ± 4.7	174.4 ± 6.2	173.8	174.2
Stride length (m)	1.19 ± 0.01	1.23 ± 0.09	1.38 ± 0.03^#$^	1.34	1.36
Peak HR (bpm)	179.3 ± 5.8	176.5 ± 4.2^#^	180.2 ± 1.6	182.0	184.0
Average HR (bpm)	165.2 ± 4.2	166.4 ± 2.0	168.8 ± 3.8	163.0	162.0
Elevation Gain (m)	249.1 ± 108.0	125.4 ± 68.6	66.9 ± 73.4	21.3	21.3
Adjusted Gain (m·min^−1^)*	4.5 ± 1.6	3.2 ± 1.7	2.0 ± 2.1	3.3	4.4
Blood Lactate (mM)	--	--	--	5.5	6.1
RPE	--	--	--	10	10

* Adjusted gain = meters gained relative to duration of the bike or run. ^#^ Significant difference from CXT, ^$^ Significant difference from RXT.

## Data Availability

Data are provided in the supplementary section of the paper.

## Supplementary Materials

The following are available online at https://www.mdpi.com/article/10.3390/sports9100136/s1, Figure S1: Graphical presentation of yearly HR TRIMP training load calculated based on a simplified 3-phase TRIMP score was calculated by multiplying the total duration in each HR zone by 1 for Zone 1, 2 for Zone 2, and 3 for Zone 3, then totaling the result, Table S1: Training summary data for a make masters off-road triathlete. Total training hours are broken down into relative sport training. Percent time in HR zones is for cycling and running combined; no HR was recorded during swimming. Approximately 20% of cycling took place mountain biking, Table S2: Results reports for ordinary least squares regression analysis for cycling and running.
