# Supplementary material for: Racing Demands of Off-Road Triathlon: A Case Study of a National Champion Masters Triathlete
Source: Sports (Basel). 2021 Sep 30;9(10):136. doi: 10.3390/sports9100136 (PMC8539484; doi:10.3390/sports9100136)
Supplement: Supplementary file 1 [file sports-09-00136-s001.zip › sports-1360721-supplementary.pdf]

Table S1. Training summary data for a make masters off-road triathlete. Total training hours are broken down into relative sport training. Percent time in HR zones is for cycling and running combined; no HR was recorded during swimming. Approximately 20% of cycling took place mountain biking.

	2015	2016	2017	2018	2019
Total Training (hrs)	470.5	461.3	482.6	467.0	456.7
**Training Discipline**					
Swim	17.0%	18.1%	13.9%	12.4%	13.7%
Bike	50.4%	48.1%	53.6%	52.6%	49.2%
Run	28.4%	29.3%	28.8%	30.8%	31.5%
Strength Training	4.3%	4.4%	3.6%	4.2%	5.6%
**Heart Rate Zones**					
Zone 1	70.3%	72.8%	77.1%	73.5%	76.6%
Zone 2	24.4%	22.3%	19.3%	22.4%	19.6%
Zone 3	5.3%	4.9%	3.7%	4.1%	4.4%

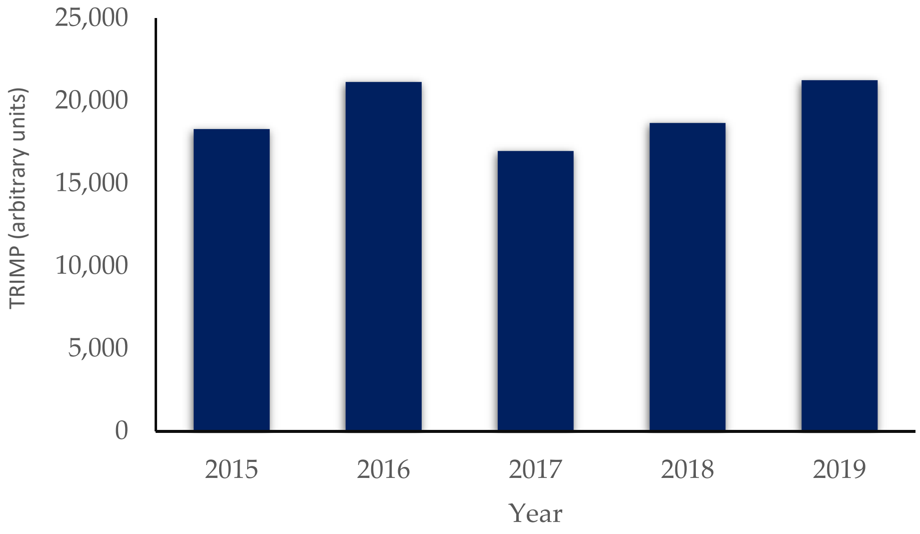

Figure S1. Graphical presentation of yearly HR TRIMP training load calculated based on a simplified 3-phase TRIMP score was calculated by multiplying the total duration in each HR zone by 1 for Zone 1, 2 for Zone 2, and 3 for Zone 3, then totaling the result.[10]

Table S2. Results reports for ordinary least squares regression analysis for cycling and running.

Events are significantly different, there does not appear to be differences between CXT and RXT; ROAD is quicker.

CXT is significantly less work/min than the road, and not significantly different than the regional, even when adjusting for elevation gain and time, which significantly impact the kj/min work rate with elevation(+) and time(-).

```
Source |        SS           df       MS      Number of obs   =        22
-------------+----------------------------------   F(4, 17)        =     29.59
       Model |  37.0515808         4  9.2628952   Prob > F        =    0.0000
```

```
     Residual |  5.32165091        17  .313038289   R-squared       =    0.8744
-------------+----------------------------------   Adj R-squared   =    0.8449
        Total |  42.3732317        21  2.01777294   Root MSE        =     .5595

----------------------------------------------------------------------------------
    kj_min_bike |      Coef.   Std. Err.      t    P>|t|     [95% Conf. Interval]
-----------------+----------------------------------------------------------------
       type_idx |
          Road  |   2.154376   .6682013     3.22   0.005     .7445946    3.564157
           RXT  |  -.7531075   .5460656    -1.38   0.186    -1.905205    .3989903
                |
bike_m_elev_gain |   .0031823   .0015168     2.10   0.051     -.000018    .0063825
       biketime |  -.0514029   .0125508    -4.10   0.001    -.0778827    -.024923
          _cons |   14.47173   1.129722    12.81   0.000     12.08823    16.85524
```

```
. reg sp_run_min_km  i.type_idx  run_m_gained

      Source |       SS           df       MS      Number of obs   =        22
-------------+----------------------------------   F(3, 18)        =      8.70
       Model |   3.7921042         3  1.26403473   Prob > F        =    0.0009
    Residual |  2.61553768        18  .145307649   R-squared       =    0.5918
-------------+----------------------------------   Adj R-squared   =    0.5238
       Total |  6.40764188        21  .305125804   Root MSE        =    .38119

------------------------------------------------------------------------------
sp_run_min~m |      Coef.   Std. Err.      t    P>|t|     [95% Conf. Interval]
-------------+----------------------------------------------------------------
    type_idx |
       Road  |  -.6299435   .3021137    -2.09   0.052    -1.264661     .004774
        RXT  |    -.23836   .2343534    -1.02   0.323    -.7307183    .2539983
             |
run_m_gained |   .0023649   .0010698     2.21   0.040     .0001173    .0046124
       _cons |   4.663165   .3085756    15.11   0.000     4.014871    5.311458
------------------------------------------------------------------------------
```

Time doesn't matter generally, but it's probably lack of data/collinearity.

```
. reg sp_run_min_km  i.type_idx  run_m_gained total_time

      Source |       SS           df       MS      Number of obs   =        22
-------------+----------------------------------   F(4, 17)        =      6.71
       Model |  3.92351246         4  .980878115   Prob > F        =    0.0020
    Residual |  2.48412942        17   .14612526   R-squared       =    0.6123
```

```
-------------+----------------------------------   Adj R-squared   =    0.5211
       Total |  6.40764188        21  .305125804   Root MSE        =    .38226

------------------------------------------------------------------------------
sp_run_min~m |      Coef.   Std. Err.      t    P>|t|     [95% Conf. Interval]
-------------+----------------------------------------------------------------
    type_idx |
       Road  |   -.387518   .3964067    -0.98   0.342    -1.223863     .448827
        RXT  |   .0023654   .3459324     0.01   0.995    -.7274882    .7322191
             |
run_m_gained |   .0017286   .0012654     1.37   0.190    -.0009411    .0043982
  total_time |   .0047316   .0049895     0.95   0.356    -.0057953    .0152585
       _cons |    3.90065   .8615682     4.53   0.000       2.0829      5.7184
------------------------------------------------------------------------------

. reg sp_run_min_km  d_road  run_m_gained time_run

      Source |       SS           df       MS      Number of obs   =        22
-------------+----------------------------------   F(3, 18)        =      8.59
       Model |  3.77207035         3  1.25735678   Prob > F        =    0.0009
    Residual |  2.63557153        18   .14642064   R-squared       =    0.5887
-------------+----------------------------------   Adj R-squared   =    0.5201
       Total |  6.40764188        21  .305125804   Root MSE        =    .38265

------------------------------------------------------------------------------
sp_run_min~m |      Coef.   Std. Err.      t    P>|t|     [95% Conf. Interval]
-------------+----------------------------------------------------------------
      d_road |  -.3298868   .2320773    -1.42   0.172     -.817463    .1576895
run_m_gained |   .0020706   .0013089     1.58   0.131    -.0006794    .0048205
    time_run |   .0128292   .0136005     0.94   0.358    -.0157443    .0414027
       _cons |   3.992165   .4719277     8.46   0.000     3.000682    4.983648
------------------------------------------------------------------------------

. reg sp_run_min_km  d_road   time_run

      Source |       SS           df       MS      Number of obs   =        22
-------------+----------------------------------   F(2, 19)        =     10.78
       Model |  3.40568127         2  1.70284064   Prob > F        =    0.0007
    Residual |  3.00196061        19  .157997927   R-squared       =    0.5315
-------------+----------------------------------   Adj R-squared   =    0.4822
       Total |  6.40764188        21  .305125804   Root MSE        =    .39749
```

```
------------------------------------------------------------------------------
sp_run_min~m |      Coef.   Std. Err.      t    P>|t|     [95% Conf. Interval]
-------------+----------------------------------------------------------------
      d_road |   -.324477   .2410516    -1.35   0.194    -.8290038    .1800498
    time_run |   .0286576   .0095686     2.99   0.007     .0086302     .048685
       _cons |   3.643277   .4333994     8.41   0.000     2.736162    4.550392
------------------------------------------------------------------------------
```

```
. reg sp_run_min_km  d_road   total_time

      Source |       SS           df       MS      Number of obs   =        22
-------------+----------------------------------   F(2, 19)        =     12.57
       Model |  3.6495262         2  1.8247631   Prob > F        =    0.0003
    Residual | 2.75811568        19  .145163983   R-squared       =    0.5696
-------------+----------------------------------   Adj R-squared   =    0.5242
       Total | 6.40764188        21  .305125804   Root MSE        =      .381

------------------------------------------------------------------------------
sp_run_min~m |      Coef.   Std. Err.      t    P>|t|     [95% Conf. Interval]
-------------+----------------------------------------------------------------
      d_road |  -.4604397   .2081883    -2.21   0.039    -.8961828   -.0246965
  total_time |   .0080151   .0023694     3.38   0.003     .0030558    .0129744
       _cons |   3.698715   .3694625    10.01   0.000     2.925421    4.472009
------------------------------------------------------------------------------
```

Total kj on bike increases min/km, but probably collinear with total run time as longer is longer for both.

```
. reg sp_run_min_km  d_road    energy_kj

      Source |       SS           df       MS      Number of obs   =        22
-------------+----------------------------------   F(2, 19)        =     12.41
       Model | 3.62939031         2  1.81469516   Prob > F        =    0.0004
    Residual | 2.77825156        19  .146223767   R-squared       =    0.5664
-------------+----------------------------------   Adj R-squared   =    0.5208
       Total | 6.40764188        21  .305125804   Root MSE        =    .38239

------------------------------------------------------------------------------
sp_run_min~m |      Coef.   Std. Err.      t    P>|t|     [95% Conf. Interval]
-------------+----------------------------------------------------------------
      d_road |  -.6556956    .195411    -3.36   0.003    -1.064696   -.2466957
   energy_kj |   .0011956   .0003569     3.35   0.003     .0004486    .0019426
       _cons |   3.839424   .3324154    11.55   0.000     3.143671    4.535177
```

```
------------------------------------------------------------------------------
```

The faster you run uphill the slower your overall run pace is on avg.

```
. reg sp_run_min_km  d_road mtr_min_run

      Source |       SS           df       MS      Number of obs   =        22
-------------+----------------------------------   F(2, 19)        =     10.05
       Model |  3.29464206         2  1.64732103   Prob > F        =    0.0011
    Residual |  3.11299982        19  .163842096   R-squared       =    0.5142
-------------+----------------------------------   Adj R-squared   =    0.4630
       Total |  6.40764188        21  .305125804   Root MSE        =    .40477

------------------------------------------------------------------------------
sp_run_min~m |      Coef.   Std. Err.      t    P>|t|     [95% Conf. Interval]
-------------+----------------------------------------------------------------
      d_road |  -.4873502   .2214607    -2.20   0.040    -.9508727   -.0238276
 mtr_min_run |    .140528   .0497711     2.82   0.011     .0363559       .2447
       _cons |   4.396699   .2062267    21.32   0.000     3.965061    4.828336
------------------------------------------------------------------------------
. reg sp_run_min_km  d_road   time_run energy_kj

      Source |       SS           df       MS      Number of obs   =        22
-------------+----------------------------------   F(3, 18)        =      7.97
       Model |  3.65603407         3  1.21867802   Prob > F        =    0.0014
    Residual |  2.75160781        18  .152867101   R-squared       =    0.5706
-------------+----------------------------------   Adj R-squared   =    0.4990
       Total |  6.40764188        21  .305125804   Root MSE        =    .39098

------------------------------------------------------------------------------
sp_run_min~m |      Coef.   Std. Err.      t    P>|t|     [95% Conf. Interval]
-------------+----------------------------------------------------------------
      d_road |  -.5617226   .3009772    -1.87   0.078    -1.194052    .0706069
    time_run |   .0078448   .0187905     0.42   0.681    -.0316327    .0473222
   energy_kj |   .0009323   .0007285     1.28   0.217    -.0005983    .0024629
       _cons |    3.72846   .4314695     8.64   0.000     2.821976    4.634943
```

W' variability is strongly collinear with elevation -- pct W' doesn't make much difference. ie, Repeated sprint efforts do not seem to relate to subsequent running performance.

```
. reg energy_kj d_road w_prime_kj biketime bike_m_elev_gain

      Source |       SS           df       MS      Number of obs   =        22
-------------+----------------------------------   F(4, 17)        =    180.75
       Model |  1131638.98         4  282909.744   Prob > F        =    0.0000
    Residual |   26607.796        17  1565.16447   R-squared       =    0.9770
-------------+----------------------------------   Adj R-squared   =    0.9716
       Total |  1158246.77        21  55154.6082   Root MSE        =    39.562

----------------------------------------------------------------------------------
       energy_kj |      Coef.   Std. Err.      t    P>|t|     [95% Conf. Interval]
-----------------+----------------------------------------------------------------
          d_road |   181.6988   43.56706     4.17   0.001     89.78033    273.6173
      w_prime_kj |   .1588444   .5026958     0.32   0.756     -.901751     1.21944
        biketime |   8.526122   .8127772    10.49   0.000     6.811312    10.24093
bike_m_elev_gain |   .1990108   .1080118     1.84   0.083    -.0288743    .4268958
           _cons |   95.59103   46.84167     2.04   0.057    -3.236261    194.4183
----------------------------------------------------------------------------------

. reg energy_kj d_road w_prime_kj biketime

      Source |       SS           df       MS      Number of obs   =        22
-------------+----------------------------------   F(3, 18)        =    211.71
       Model |   1126325.6         3  375441.867   Prob > F        =    0.0000
    Residual |  31921.1713        18  1773.39841   R-squared       =    0.9724
-------------+----------------------------------   Adj R-squared   =    0.9678
       Total |  1158246.77        21  55154.6082   Root MSE        =    42.112

------------------------------------------------------------------------------
   energy_kj |      Coef.   Std. Err.      t    P>|t|     [95% Conf. Interval]
-------------+----------------------------------------------------------------
      d_road |   157.1305   44.14926     3.56   0.002     64.37631    249.8846
  w_prime_kj |    .290721   .5296403     0.55   0.590     -.822012    1.403454
    biketime |   9.488405   .6629001    14.31   0.000     8.095704    10.88111
       _cons |   81.83176   49.22266     1.66   0.114    -21.58121    185.2447
------------------------------------------------------------------------------
```

```
. reg energy_kj d_road w_prime_kj

      Source |       SS           df       MS      Number of obs   =        22
-------------+----------------------------------   F(2, 19)        =     18.34
       Model |  762999.506         2  381499.753   Prob > F        =    0.0000
    Residual |  395247.266        19  20802.4877   R-squared       =    0.6588
-------------+----------------------------------   Adj R-squared   =    0.6228
       Total |  1158246.77        21  55154.6082   Root MSE        =    144.23

------------------------------------------------------------------------------
   energy_kj |      Coef.   Std. Err.      t    P>|t|     [95% Conf. Interval]
-------------+----------------------------------------------------------------
      d_road |   532.2771   121.6812     4.37   0.000     277.5955    786.9587
  w_prime_kj |   6.420465    1.06736     6.02   0.000     4.186454    8.654475
       _cons |  -72.43468   164.4944    -0.44   0.665    -416.7254    271.8561
------------------------------------------------------------------------------

. corr energy_kj d_road w_prime_kj biketime bike_m_elev_gain
(obs=22)

             | energy~j   d_road w_prim~j biketime bike~ain
-------------+---------------------------------------------
   energy_kj |   1.0000
      d_road |  -0.0943   1.0000
  w_prime_kj |   0.5613  -0.7977   1.0000
    biketime |   0.9575  -0.3302   0.7236   1.0000
bike_m_e~ain |   0.7451  -0.6133   0.8373   0.8354   1.0000

. reg sp_run_min_km run_m_gained energy_kj i.type_idx

      Source |       SS           df       MS      Number of obs   =        22
-------------+----------------------------------   F(4, 17)        =      6.79
       Model |  3.94123883         4  .985309708   Prob > F        =    0.0019
    Residual |  2.46640305        17  .145082532   R-squared       =    0.6151
-------------+----------------------------------   Adj R-squared   =    0.5245
       Total |  6.40764188        21  .305125804   Root MSE        =     .3809

------------------------------------------------------------------------------
sp_run_min~m |      Coef.   Std. Err.      t    P>|t|     [95% Conf. Interval]
-------------+----------------------------------------------------------------
run_m_gained |   .0017803   .0012145     1.47   0.161    -.0007821    .0043428
   energy_kj |   .0007175   .0007077     1.01   0.325    -.0007756    .0022107
```

```
             |
    type_idx |
       Road  |  -.4920969   .3310841    -1.49   0.156    -1.190623    .2064295
        RXT  |   .0097676   .3387196     0.03   0.977    -.7048682    .7244034
             |
       _cons |   3.959658   .7593055     5.21   0.000     2.357664    5.561653
```

```
. reg run_pace run_m_gained

      Source |       SS           df       MS      Number of obs   =        22
-------------+----------------------------------   F(1, 20)        =     18.75
       Model |  8.02958879         1  8.02958879   Prob > F        =    0.0003
    Residual |  8.56613643        20  .428306821   R-squared       =    0.4838
-------------+----------------------------------   Adj R-squared   =    0.4580
       Total |  16.5957252        21  .790272629   Root MSE        =    .65445

------------------------------------------------------------------------------
    run_pace |      Coef.   Std. Err.      t    P>|t|     [95% Conf. Interval]
-------------+----------------------------------------------------------------
run_m_gained |   .0059519   .0013746     4.33   0.000     .0030845    .0088194
       _cons |   6.769432   .2442611    27.71   0.000     6.259912    7.278951
------------------------------------------------------------------------------
```

```
. reg run_pace time_run

      Source |       SS           df       MS      Number of obs   =        22
-------------+----------------------------------   F(1, 20)        =     18.97
       Model |  8.07920445         1  8.07920445   Prob > F        =    0.0003
    Residual |  8.51652077        20  .425826038   R-squared       =    0.4868
-------------+----------------------------------   Adj R-squared   =    0.4612
       Total |  16.5957252        21  .790272629   Root MSE        =    .65255

------------------------------------------------------------------------------
    run_pace |      Coef.   Std. Err.      t    P>|t|     [95% Conf. Interval]
-------------+----------------------------------------------------------------
    time_run |   .0574018   .0131782     4.36   0.000     .0299125    .0848912
       _cons |   5.281566   .5584796     9.46   0.000     4.116598    6.446534
------------------------------------------------------------------------------
```

```
. reg run_pace time_run run_m_gained

      Source |       SS           df       MS      Number of obs   =        22
```

```
-------------+----------------------------------   F(2, 19)        =     11.27
       Model |  9.00338531         2  4.50169266   Prob > F        =    0.0006
    Residual |   7.5923399        19  .399596837   R-squared       =    0.5425
-------------+----------------------------------   Adj R-squared   =    0.4944
       Total |  16.5957252        21  .790272629   Root MSE        =    .63214

------------------------------------------------------------------------------
    run_pace |      Coef.   Std. Err.      t    P>|t|     [95% Conf. Interval]
-------------+----------------------------------------------------------------
    time_run |   .0324514   .0207879     1.56   0.135    -.0110581    .0759609
run_m_gained |   .0032881   .0021621     1.52   0.145    -.0012373    .0078135
       _cons |   5.826042   .6487438     8.98   0.000     4.468206    7.183878
------------------------------------------------------------------------------

. reg run_pace time_run energy_kj

      Source |       SS           df       MS      Number of obs   =        22
-------------+----------------------------------   F(2, 19)        =      9.04
       Model |  8.09001374         2  4.04500687   Prob > F        =    0.0017
    Residual |  8.50571147        19  .447669025   R-squared       =    0.4875
-------------+----------------------------------   Adj R-squared   =    0.4335
       Total |  16.5957252        21  .790272629   Root MSE        =    .66908

------------------------------------------------------------------------------
    run_pace |      Coef.   Std. Err.      t    P>|t|     [95% Conf. Interval]
-------------+----------------------------------------------------------------
    time_run |   .0548339   .0213464     2.57   0.019     .0101555    .0995124
   energy_kj |   .0001526   .0009822     0.16   0.878    -.0019031    .0022083
       _cons |   5.252247   .6029082     8.71   0.000     3.990346    6.514148
------------------------------------------------------------------------------

. reg run_pace time_run biketime

      Source |       SS           df       MS      Number of obs   =        22
-------------+----------------------------------   F(2, 19)        =     10.05
       Model |  8.52937293         2  4.26468646   Prob > F        =    0.0011
    Residual |  8.06635229        19  .424544857   R-squared       =    0.5139
-------------+----------------------------------   Adj R-squared   =    0.4628
       Total |  16.5957252        21  .790272629   Root MSE        =    .65157

------------------------------------------------------------------------------
    run_pace |      Coef.   Std. Err.      t    P>|t|     [95% Conf. Interval]
```

```
-------------+----------------------------------------------------------------
    time_run |   .0384365   .0226353     1.70   0.106    -.0089396    .0858126
    biketime |   .0100908   .0097994     1.03   0.316    -.0104196    .0306011
       _cons |   5.286414   .5576587     9.48   0.000     4.119221    6.453607
------------------------------------------------------------------------------

. reg run_pace time_run i.type_idx

      Source |       SS           df       MS      Number of obs   =        22
-------------+----------------------------------   F(3, 18)        =      7.20
       Model |  9.05093051         3  3.01697684   Prob > F        =    0.0022
    Residual |  7.54479471        18  .419155262   R-squared       =    0.5454
-------------+----------------------------------   Adj R-squared   =    0.4696
       Total |  16.5957252        21  .790272629   Root MSE        =    .64742

------------------------------------------------------------------------------
    run_pace |      Coef.   Std. Err.      t    P>|t|     [95% Conf. Interval]
-------------+----------------------------------------------------------------
    time_run |   .0348155   .0218066     1.60   0.128    -.0109985    .0806296
             |
    type_idx |
       Road  |  -.8976722   .6409368    -1.40   0.178     -2.24423     .448886
        RXT  |  -.3407455   .4597451    -0.74   0.468    -1.306634    .6251431
             |
       _cons |   6.582961   1.200486     5.48   0.000     4.060834    9.105088
```
